# Rare Species Support Vulnerable Functions in High-Diversity Ecosystems

**DOI:** 10.1371/journal.pbio.1001569

**Published:** 2013-05-28

**Authors:** David Mouillot, David R. Bellwood, Christopher Baraloto, Jerome Chave, Rene Galzin, Mireille Harmelin-Vivien, Michel Kulbicki, Sebastien Lavergne, Sandra Lavorel, Nicolas Mouquet, C. E. Timothy Paine, Julien Renaud, Wilfried Thuiller

**Affiliations:** 1 Unité Mixte de Recherche (UMR) Centre National de la Recherche Scientifique (CNRS)–UM2– Institut français de recherche pour l'exploitation de la mer (IFREMER)–Institute for Research and Development (IRD) 5119 ECOSYM, Université Montpellier 2 cc 093, Montpellier, France; 2 ARC Centre of Excellence for Coral Reef Studies, James Cook University, Townsville, Australia; 3 School of Marine and Tropical Biology, James Cook University, Townsville, Australia; 4 Institut National de la Recherche Agronomique, UMR Ecologie des Forêts de Guyane, 97387, Kourou, French Guiana, France; 5 Department of Biology, University of Florida, Gainesville, Florida, United States of America; 6 Université Paul Sabatier, CNRS, Laboratoire Evolution et Diversité Biologique UMR 5174, Toulouse, France; 7 Laboratoire d'Excellence “Corail” USR 3278 CNRS-EPHE, BP 1013, Moorea, Polynésie Française, France; 8 Aix-Marseille Université, Institut Méditerranéen d'Océanologie (MIO), UMR CNRS 7294, Marseille, France; 9 Laboratoire d'Excellence “Corail” IRD, Laboratoire Arago, Banyuls/mer, France; 10 Laboratoire d'Ecologie Alpine, UMR CNRS 5553, Université Joseph Fourier, BP53, Grenoble, France; 11 Institut des Sciences de l'Evolution, UMR CNRS–UM2, 5554, Université Montpellier 2, Montpellier, France; 12 Biological and Environmental Sciences, University of Stirling, Stirling, United Kingdom; University College London, United Kingdom

## Abstract

Around the world, the human-induced collapses of populations and species have triggered a sixth mass extinction crisis, with rare species often being the first to disappear. Although the role of species diversity in the maintenance of ecosystem processes has been widely investigated, the role of rare species remains controversial. A critical issue is whether common species insure against the loss of functions supported by rare species. This issue is even more critical in species-rich ecosystems where high functional redundancy among species is likely and where it is thus often assumed that ecosystem functioning is buffered against species loss. Here, using extensive datasets of species occurrences and functional traits from three highly diverse ecosystems (846 coral reef fishes, 2,979 alpine plants, and 662 tropical trees), we demonstrate that the most distinct combinations of traits are supported predominantly by rare species both in terms of local abundance and regional occupancy. Moreover, species that have low functional redundancy and are likely to support the most vulnerable functions, with no other species carrying similar combinations of traits, are rarer than expected by chance in all three ecosystems. For instance, 63% and 98% of fish species that are likely to support highly vulnerable functions in coral reef ecosystems are locally and regionally rare, respectively. For alpine plants, 32% and 89% of such species are locally and regionally rare, respectively. Remarkably, 47% of fish species and 55% of tropical tree species that are likely to support highly vulnerable functions have only one individual per sample on average. Our results emphasize the importance of rare species conservation, even in highly diverse ecosystems, which are thought to exhibit high functional redundancy. Rare species offer more than aesthetic, cultural, or taxonomic diversity value; they disproportionately increase the potential breadth of functions provided by ecosystems across spatial scales. As such, they are likely to insure against future uncertainty arising from climate change and the ever-increasing anthropogenic pressures on ecosystems. Our results call for a more detailed understanding of the role of rarity and functional vulnerability in ecosystem functioning.

## Abstract 2

The most unusual, and thus irreplaceable, functions performed by species in three different species-rich ecosystems are fulfilled by only the rare species in these ecosystems.

## Author Summary

In ecological systems most species are rare—that is, represented by only a few individuals or restricted to particular habitats—and are vulnerable to being lost. Yet the ecological consequences of such biodiversity loss are often overlooked and remain controversial. In the best-case scenario, the functions that these rare species provide to their ecosystems might be insured by more common species, which share combinations of functional traits with the rare species, thereby helping to maintain ecosystem functioning despite rare species loss. In the worst-case scenario, rare species would have functional traits that are distinct from those of common species; thus, the functions they support would also be vulnerable to extinction. We examined three highly diverse ecosystems (coral reefs, alpine meadows, and tropical forests) and addressed whether common species would insure against the loss of functions carried by rare species. We demonstrate that highly distinct combinations of traits are supported predominantly by rare species. It is thus not only the quantity but also the quality of biodiversity that matters. Thus, our findings highlight that we need to change how we think about biodiversity in general, and about conservation strategies in particular, by moving beyond the protection of biodiversity per se and beyond focusing on iconic, charismatic, or phylogenetically distinct species, to protecting species that support irreplaceable functional roles and associated services.

## Introduction

The vast majority of species are rare—that is, comprising few individuals—and often have restricted geographic distributions [1]. Although several forms of rarity have been defined with respect to the trajectories by which species become extinct [2],[3], rare species are all seen as highly vulnerable to overexploitation [4], habitat loss [5], competitive interactions with exotic species [6], and climate change [7]. Rare species have thus received important consideration from conservation biologists because their extirpation contributes disproportionately to the ongoing sixth extinction crisis [8]. This biotic impoverishment may, in turn, alter the biogeochemical and dynamic properties of ecosystems [9]. Beyond aesthetic, cultural, and moral arguments, the maintenance of ecosystem functioning has thus become a powerful justification to limit biodiversity erosion [10]. Indeed, most key ecosystem processes, such as organic matter degradation, bioturbation, bioerosion, and productivity, are threatened by the loss of functions performed by particular species [11],[12],[13], some of which may be rare.

It has long been assumed that the loss of rare species will have a limited impact on ecosystem functioning at short terms and local scales, given their low abundance within communities [14]. However, this hypothesis has been challenged because the loss of rare species can affect local ecosystem processes [15],[16] and rare species can contribute significantly to long-term and large-scale ecosystem functioning [17], eventually providing ecological insurance in variable environments where species abundances vary in time [18]. Indeed, rare species may perform functions complementary to those delivered by other, even closely related, species as a result of their distinct functional traits [19]. In turn, those rare species may increase the functional diversity of local communities [20], sustain ecosystem functioning [21], and provide functional traits able to support the main ecosystem processes under future environmental conditions [18].

Ecosystems depend on the maintenance of multiple processes [13] across space and time under environmental-change scenarios [22]. This requires species with complementary functions [23]; however, current knowledge is still far from being able to assess the roles played by individual species, especially in highly diverse regions where data are lacking even for common species. Rather, current practice is to assess the ecological role of species indirectly via their functional traits. Here, we assume that species with distinct combinations of functional traits are more likely to support functions that cannot be delivered by species with more-common traits. This assumption is based on experiments showing that species with traits that are not present in others (thus minimizing functional redundancy) regulate ecosystem processes [24], and that trait dissimilarity within species communities, favored by the presence of species with distinct trait combinations, increases ecological process rates [21],[25]. A modeling study further showed that the covariance between species extinction risks and their functional traits mediates bioturbation, with species possessing the most distinct traits having the highest impact [12]. In practice, this assumption needs to consider multiple functional traits to embrace the range of potential roles that species may play [26]. In this respect, some species play unique roles in the ecosystem according to the distinctiveness of their functional traits relative to the rest of the species pool [27]. The loss of species with such distinctive traits may thus affect ecosystem functioning [12], especially when multiple functions are considered [21]. Conversely, functional redundancy, where different species sustain similar functions, may insure against the loss of ecosystem functioning following biodiversity erosion [28],[29]. It is therefore critical to know the degree to which rare species share combinations of functional traits with common species.

In the best-case scenario, common species would share combinations of functional traits with rare species, thereby maintaining ecosystem functioning despite the loss of rare species. The protection of common species would thus become the primary focus for the maintenance of ecosystem processes [30]. In the worst-case scenario, rare species would have functional traits markedly distinct from those of common species; hence the functions they support would be vulnerable to extinction. Vulnerable functions are, therefore, defined by having low insurance—that is, there are few species and few individuals with similar combinations of traits that provide this particular function. In this case, the loss of rare species would have greater ecosystem impacts than expected simply as a result of numerical species loss. The conservation of rare species would thus be a priority for the maintenance of ecosystem functioning, beyond the classic motivations of preserving the diversity of life and the precautionary principle [31]. This issue is even more critical in species-rich ecosystems where high functional redundancy among species is likely [32],[33] and where it is thus often assumed that ecosystem functioning is buffered against species loss.

Recent studies that investigated the contribution of rare species to functional diversity reached inconsistent conclusions, but were restricted to local samples of a limited number of species [20],[34]–[36]. The question of whether species with unusual combinations of functional traits, which are likely to support vulnerable ecological functions, are overwhelmingly rare is still unresolved in species-rich regional assemblages and at large scales. An extensive body of literature has looked at why some species are specialists and searched for suites of traits underpinning the link between rarity and specialization [37]. In our study, we adopted an alternative approach by focusing on whether distinct trait combinations, which could be irreplaceable, were likely to be supported by rare species. Using extensive datasets of species local abundances, regional occurrences, and functional traits from three highly diverse ecosystems (846 coral reef fishes, 2,979 alpine plants, and 662 tropical trees), we demonstrate that highly distinct combinations of traits are supported predominantly by rare species both at the local and regional scales. Moreover, we show that the species that are likely to support the most vulnerable functions—that is, those that might be supported by poorly insured functional trait values—are rarer than expected by chance in all three ecosystems, again at both local and regional scales.

## Results

For each of the three datasets we estimated two complementary aspects of rarity: (*i*) local abundance as the abundance in communities where the species was found and (*ii*) regional occupancy as the proportion of communities in which the species was recorded. For simplicity, we use “common” as the antithesis of “rare” regardless of the scale considered. Rarity is a continuous measure, so we defined two thresholds to classify species. At a local scale, we defined “rare” species as those with a local abundance (number of individuals for fish and trees, surface cover for plants) less than 5% of the most abundant species, whereas the “rarest” species were those represented by a single individual (for fish and trees) or less than 1% of most abundant species (for plants). At the regional scale, we defined “rare” and “rarest” species as those having less than 5% of the occupancy of the most common species in the dataset, and as those having only one occurrence, respectively. We estimated the functional distinctiveness of each species using its functional distance from the rest of the species pool based on multiple traits. We then regressed functional distinctiveness against regional occupancy, both being measured on a standardized scale to allow comparisons among ecosystems.

**Figure 1:**
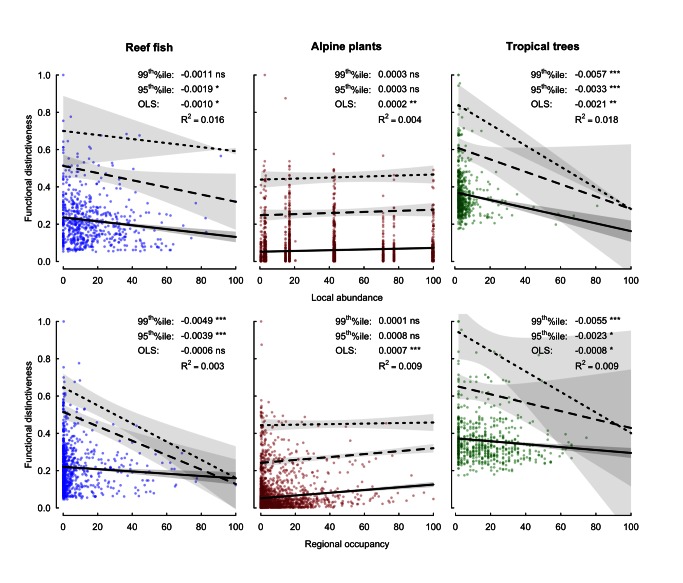
Functional distinctiveness as a function of commonness. Species commonness is measured at the local scale as the mean abundance over all the samples where the species is present and expressed as a percentage of the maximum observed value, and at the regional scale, it is measured as the number of occurrences over all the samples and expressed as a percentage of the maximum observed value. Functional distinctiveness, expressed as a proportion of the maximum observed value, quantifies the uniqueness of species biological traits from the rest of the pool in the ecosystem. Solid lines represent ordinary least square regressions, whereas dashed and dotted lines represent 95^th^ and 99^th^ quantile regressions, respectively. Shaded areas indicate the 95% confidence intervals for each relationship. All variables are standardized to allow comparisons among ecosystems and spatial scales. *^ns^ p*>0.05, * *p*≤0.05, ** *p*≤0.01, *** *p*≤0.001.

Functional distinctiveness was negatively and significantly related to commonness, whether estimated as local abundance or regional occupancy (Figure 1). Reef fishes and tropical trees show a consistently triangular relationship: the most unusual combinations of functional traits—that is, those with high functional distinctiveness—were invariably supported by rare species, whereas species with low functional distinctiveness were either common or rare. For alpine plants, the slopes of the 95^th^ and 99^th^ quantile regressions were not significant at both scales, but the two species with the highest functional distinctiveness values (*Saxifraga mutata* and *Rosa sempervirens*) were rare at local and regional scales. Across all three ecosystems, the most functionally distinct species (having a functional distinctiveness value higher than that predicted by the 99^th^ quantile regression) all had a regional occupancy less than 50% of the maximum value and most of them were rare (Figure 1).

**Figure 2:**
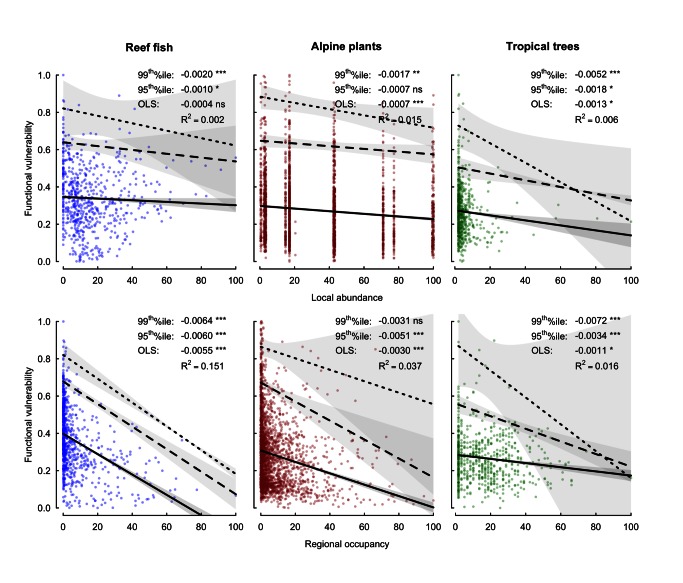
Functional vulnerability as a function of commonness. Species commonness is measured as in Figure 1. Functional vulnerability, scaled between 0 and 1, quantifies the lack of functional insurance provided by the rest of the pool to the focal species in terms of functional traits and regional occupancy. Solid lines represent ordinary least square regressions, whereas dashed and dotted lines represent 95^th^ and 99^th^ quantile regressions, respectively. Shaded areas indicate the 95% confidence intervals for each relationship. *^ns^ p*>0.05, * *p*≤0.05, ** *p*≤0.01, *** *p*≤0.001.

We then estimated the potential vulnerability of the functions supported by each species. Vulnerability is inversely related to the extent of insurance provided by functionally similar common species. If a species shares a similar combination of traits with common species, it is more likely to support functions with a high insurance and low vulnerability to extinction. Vulnerability is therefore estimated based on the commonness of species that share similar combinations of traits. At both scales, in all three ecosystems, functional vulnerability significantly decreased with commonness, resulting in concordant triangular relationships (Figure 2). The most vulnerable functions, those that might be supported by poorly insured combinations of functional traits, were mainly supported by rare species, whereas common species never supported highly vulnerable functions.

**Figure 3:**
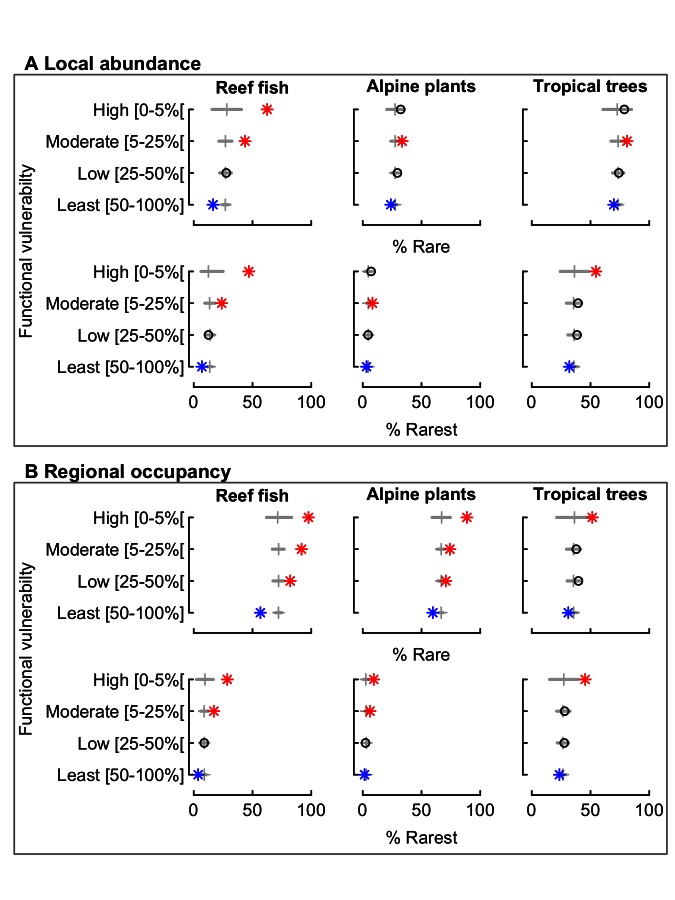
Percentage of rare and rarest species for different levels of functional vulnerability. The functional vulnerability index is scaled (0–1) and was divided into four categories from high to least. Locally rare species are those with a mean abundance value over the samples where present less than 5% of the maximum local abundance value and rarest species are those with only one individual by sample where present on average. Regionally rare species are those with less than 5% of the maximum regional occupancy value and rarest species are those with only one occurrence over all the samples. For each level of vulnerability, we obtained the confidence interval at 90% by randomization and we tested whether the observed percentage of rare and rarest species is higher (red star) or lower (blue star) than expected by chance, otherwise black circle. The vertical grey line is the median obtained at random. Sample sizes are provided in Table S1.

The association of rarity and functional vulnerability could result from a sampling effect, given the many rare species in our datasets. Therefore, we tested whether the rare or rarest species, at two different scales, were over- or underrepresented in different levels of functional vulnerability. We compared the observed percentages of rare and rarest species for different levels of functional vulnerability with those expected if rarity and functional vulnerability were independent. At the local scale (Figure 3A), the rarest species (only one individual by sample) were significantly overrepresented among reef fishes (47% against 12.5% expected) and tropical trees (54% against 36% expected) that are the most likely to support highly vulnerable functions (top 5%). Rarest species were consistently and significantly underrepresented among species supporting the least vulnerable functions (last 50%) in all three ecosystems. Rare species (less than 5% of local abundance) also contributed more than expected to the pool of species supporting highly and moderately vulnerable functions whatever the ecosystem, reaching a value up to 80% for tropical trees. At a regional scale (Figure 3B), in all three ecosystems, the rarest species were significantly overrepresented among those most likely to support highly vulnerable functions (top 5%) and underrepresented among species supporting the least vulnerable functions (last 50%). Rare species were even more overrepresented among those supporting highly vulnerable functions, whereas they were consistently underrepresented among those supporting the least vulnerable functions. For instance, 98% of fish species that were likely to support highly vulnerable functions in coral reef ecosystems were rare. This percentage was 89% and 52% for alpine plants and tropical trees, respectively.

The overrepresentation of rare and rarest species among those that support highly and moderately vulnerable functions could potentially result from the inclusion in our datasets of species from neighboring biogeographic regions. One would expect such “marginal” species to have combinations of traits adapted to other ecosystems, and to colonize only the edges of the studied ecosystems. If these “marginal” species were generating the observed rarity–vulnerability relationships (Figure 3), then we would predict that the species supporting highly and moderately vulnerable functions would occur farther from the geographic center of each ecosystem than would randomly chosen species. After calculating the marginality of each species, we performed randomization tests. They show that species supporting highly and moderately vulnerable functions were no more marginal than expected by chance (Figure S1). This result refutes the hypothesis that the most vulnerable functions were mainly supported by rare but geographically marginal species.

## Discussion

The link between species rarity and functional vulnerability is critical to understand the implications of biodiversity erosion for the decline of ecosystem functioning. Our study tackles this issue using three species-rich ecosystems at two different scales and offers a clear result: the combinations of traits with the highest distinctiveness values are all supported by rare species (Figure 1). We also assessed to what extent some “functional insurance” against the loss of rare species would be provided by regionally common species sharing similar combinations of traits. Since the relationships are triangular (Figure 2) we do not suggest that all rare species support distinct and vulnerable functions; indeed, most rare species probably support common and redundant functions. However, our results unambiguously show that rare species, those that have low local abundance and are regionally sparse, consistently carry the least-redundant combinations of traits.

If the distinctiveness of species-trait combinations does indeed map to distinct ecological functions, then such functions are likely to be the most vulnerable, given the ongoing threats to the rare species that sustain them [6]. This may be particularly important in areas with intense human impacts [5],[38]. We therefore suggest that the conservation of rare species offers more than taxonomic, aesthetic, cultural, or ethical value and must be also considered, in the addition to that of common species, when planning for the long-term maintenance of ecosystem functioning. For instance, some coral reefs can maintain processes and deliver services with a fraction of the species seen on reefs elsewhere [29],[39], but our results indicate that rare species may be functionally important and cannot be discounted. Indeed, our remarkably consistent results across scales highlight that, beyond protecting species with a low area of occupancy at a regional scale, it would be equally important to protect species that are locally rare, since they tend to support the more vulnerable functions and increase the level of functional diversity within communities, which in turn sustains local ecosystem processes [21],[40]. This latter argument is in agreement with a recent study showing that, using a global survey of reef fish assemblages, ecosystem functioning (as measured by standing biomass) scales in a non-saturating manner with biodiversity (measured either as species richness or functional diversity using the same fish traits as in our study) [41]. This precautionary principle applies in highly diverse ecosystems, characterized by high functional redundancy among species [32],[33], and even more so in lower diversity ecosystems where the potential for functional redundancy is limited [42],[43].

**Figure 4:**
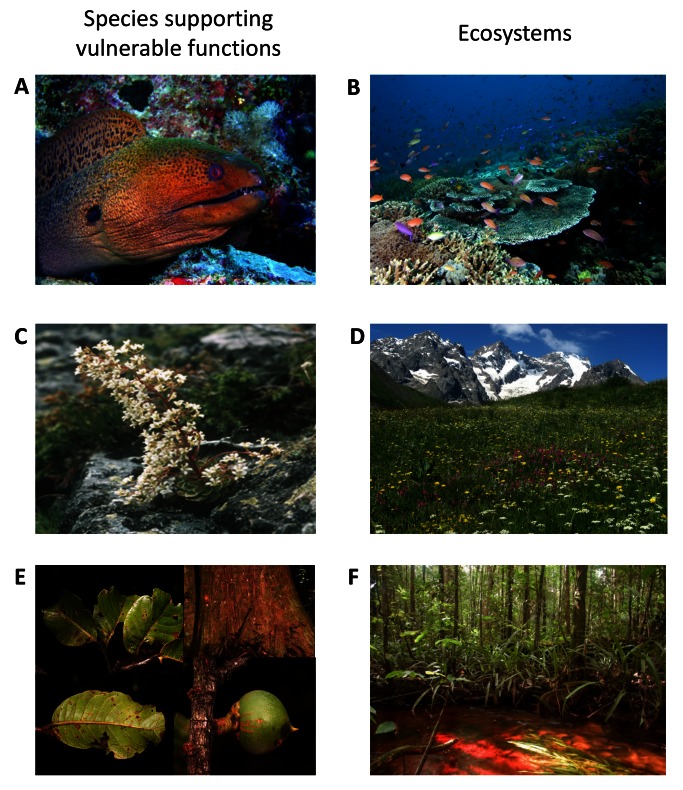
Species supporting some of the most vulnerable functions on coral reefs, in alpine meadows, and in rain forests. (A) The giant moray eel (*Gymnothorax javanicus* (Muraenidae)), the largest of the moray eels, hunts by night within the labyrinth of the coral reef (B). (C) *Saxifraga cotyledon* (Saxifragaceae) is a low-growing, rare evergreen perennial plant, with long flowering stems that make it an important resource for pollinators on species-poor siliceous alpine cliffs (D). (E) *Pouteria maxima* (Sapotaceae), a rain forest tree with thick, coriaceous leaves and a wide buttressed trunk with thick bark, which may buffer the impacts of drought and fire predicted to occur more frequently for tropical forests (F). Photo credits: (A) M.J. Kramer, (B) J.P. Krajewski, (C) J.P. Dalmas, (D) W. Thuiller, (E, F) C.E.T. Paine.

The functional importance of species carrying the most vulnerable combinations of traits is underlined by a closer examination of some of their roles in each ecosystem. On coral reefs, for example, the giant moray eel (*Gymnothorax javanicus*), ranked with the fifth-highest functional vulnerability value, is a large sedentary nocturnal benthic predator with few potential challengers to this role (Figure 4A). Likewise, the batfish (*Platax pinnatus*), supporting the 20^th^ most vulnerable function, was recently identified as a key species in reef regeneration following a phase-shift to macroalgae—a role that many common herbivorous species were unable to play [44]. For plants, some functionally distinct rare species might seem unimportant at first glance but can have critical roles. For instance, the Pyramidal Saxifrage (*Saxifraga cotyledon*), a spectacular plant inhabiting cliffs (Figure 4C), occupies the 3^rd^ rank for functional vulnerability. It has thick and dense leaves with long life span, indicative of slow plant growth and an adaptation to highly stressful environments [19]. *S. cotyledon* also possesses exceptionally long flowering stems, which makes it easy to detect and provides a locally important resource for pollinators in those species-poor habitats. *Cytisus polytrichus*, ranked 5^th^ for functional vulnerability, is one of the few myrmechorous species in the region (i.e., dispersed by ants) and thus likely to be a principal resource for ant species. Among tropical trees, *Pouteria maxima* (Sapotaceae), which has the highest functional vulnerability value, is a recently described species known only from three collections in eastern French Guiana (Figure 4E). This tree grows to more than 40 m in height and at least 75 cm in diameter, with buttresses rising to 8 m in height. Its functional distinctiveness hinges on its very thick, dense leaves coupled with very thick plate-like bark and low-density wood. These traits provide it with the potential for exceptional resilience to the increasing frequency and intensity of fires that are likely to occur in the region [45], making the species an important potential buffer maintaining both forest structure and functioning during global climate change.

Our results thus call for new approaches that will specifically address the role of rarity and functional vulnerability in ecosystem functioning with, for example, experiments using species for which we have information (abundance and traits) in controlled designs where species richness and relative abundances would be kept constant. An important step forward will be to scale up our results from the one-trait one-function perspective to a more sophisticated multifunctionality perspective [21], to disentangle the relative contribution of rare and common species traits to complex ecosystem properties. As a complementary investigation, and since the species functional traits that determine ecosystem functioning may also drive their extinction risk, the level of covariation across species between the susceptibility to decline and the contribution to ecosystem functioning needs to be known [12],[46]. The loss of vulnerable functions, those that are overwhelmingly supported by rare species, may also render communities and hence ecosystem processes more unstable in the face of fluctuating environmental stressors at longer time scales. For instance, the salinity stress may change the hierarchy of successful functional traits in phytoplankton communities and compensatory growth of rare species may sustain primary productivity [18]. The conservation and restoration of communities may thus need to maintain or re-establish both dominant species that provide high levels of target functions and rare species, which might provide additional key functions under future conditions [47]. At a longer time scale, it remains crucial to know whether observed macro-evolutionary patterns of species functional traits would lead to niche filling and recovery of functions that were lost following selective species extinctions [48].

In the end, it is the functional abilities of species that are critical in maintaining ecosystems. Our results indicate that rare species may deliver more unusual and important functions than their local abundance or regional occupancy may suggest. We also show that such species are not geographically marginal, highlighting their potential importance to resilient ecosystem functioning particularly given future environmental uncertainty. Thus, even in highly diverse systems, we can no longer assume that rare species can be discounted by the high probability of functional redundancy. In these high-diversity systems, rare species may be as important as their more common counterparts.

## Materials And Methods

### Datasets

#### Reef Fishes

Reef fish were sampled along 50 m underwater visual transects using a distance sampling method [49]. Transects were laid on reefs in less than 15 m depths. All individuals greater than 4 cm in length were identified at the species level and counted. Transects were conducted in four regions of the South Pacific, with 1,485 transects in New Caledonia, 170 in Fiji, 205 in Tonga, and 590 in French Polynesia. A total of 1,390,000 fish individuals and 846 species were recorded. The regional occupancy of each species was estimated as the number of occurrences across the transects taking into account the unequal contribution of each region. The local abundance of each species was estimated as the mean number of individuals counted across the transects where that species was present. We eliminated 213 species for which we had no confidence on abundance estimation (e.g., cryptic, schooling, or synonymy) leaving a pool of 633 fish species.

Information on six life-history traits was extracted for each species (Text S1).

#### Alpine Plants

Occurrence data for the alpine plants were compiled from the National Alpine Botanical Conservatory (CBNA) [50]. This dataset contains approximately two million spatially localized single occurrences (i.e., presence-only data and presence-absence relevés) recorded from 1980 to 2009. To homogenize the sampling—that is, to take into account the unequal density of samples—we aggregated the relevés into a 250×250 m grid resolution. As soon as a species was recorded at least once within each grid-cell, the grid-cell was given a presence of that species. The botanists of the CBNA carefully checked final maps. Regional occupancy was then estimated by summing the number of grid-cells in which the species was present for 2,979 species.

To estimate local abundance, we used a database of vegetation surveys provided by the CBNA, including 8,160 community-plots sampled in natural or seminatural areas from 1980 to 2009 and with a total of 2,535 plant species. Plot size information was not systematically available but was approximately 10×10 m. Within each community-plot, species abundances were recorded using a cover scheme with six classes (1, less than 1%; 2, from 1% to 5%; 3, from 5% to 25%; 4, from 25% to 50%; 5, from 50% to 75%; 6, up to 75%). Species abundance classes were converted to abundances using the mean percentages of the classes (0.5%, 3%, 15%, 37.5%, 62.5%, and 87.5%). We then calculated the local abundance of each species by extracting the maximum species' abundance over the plots as a measure of rarity—that is, the maximum cover the species can reach locally. The mean abundance over the plots was not retained since abundance values were not homogeneously distributed between 0% and 100% and were better interpreted as thresholds.

Ten traits were selected from the Androsace database, a functional trait database for the French Alpine Flora composed mainly from own measurements (Text S1).

#### Tropical Trees

For the tropical tree dataset, field sampling for functional traits was conducted in 2007 and 2008 at nine one-hectare plots representing a gradient of precipitation and geological substrates across lowland tropical forests in French Guiana. In each plot, all trees >10 cm diameter at 1.3 m height (dbh) were mapped and measured for height and dbh. Each tree was climbed to obtain a branch for leaf and twig samples. Herbarium vouchers were collected for every single stem to verify botanical identifications, with consultation of taxonomic specialists when necessary. These taxa represented a total of 662 species, 217 genera, and 56 families (*sensu* Angiosperm Phylogeny Group III).

To obtain a measure of regional occupancy for the species collected in French Guiana, we considered a larger biogeographical area, in which we assessed the frequency of occurrence of our censused species. Within the Guiana granitic shield, three countries cover much of the territory that does not include the Amazon and Orinoco watersheds: Guyana, Surinam, and French Guiana. These countries span a total area of 461,000 km^2^, and the floristic taxonomy has been standardized carefully through an international effort [51]. We used a compilation based on the records available in the Global Biodiversity Information Facility (GBIF) for which we were able to confirm taxonomic determinations in regional herbaria. To the primary list we added over 10,000 records from our own collections of these species in our plots. Overall our dataset included 32,491 geo-referenced collections for the 662 species included in the analysis. We recorded the occurrence (presence-absence) of each of these taxa in 478 0.25°×0.25° grid cells.

To obtain a measure of local abundance, we used a regional dataset made of permanent plots throughout French Guiana, for which taxonomic determinations have been standardized using the Cayenne regional herbarium of IRD (CAY). These datasets represent more than 44,000 trees observed across 76 ha of forest. From these data, we estimated the average relative abundance of each species as the mean density per hectare across all permanent plots in the sample in which that species was observed. For the abundance measures, we excluded three morphospecies for which we were not confident of taxonomic synonymy, leaving a total of 659 species. The mean number of plots was 66 (minimum, 1; maximum, 497 for *Eperua falcata*). The mean number of occurrences was of 25 (minimum, 1; maximum, 116, *Tapiria guianensis*). The size of the plots included in the abundance analysis was typically of 1 ha, but a few (e.g., at the Paracou station) were larger in size (6.25 ha).

For each tree, 14 leaf and trunk functional traits were measured (Text S1) representing leaf and wood economics [52]. We computed the mean trait value for each of the 662 free-standing species in the dataset.

### Rarity Estimation

Rarity can be considered at different spatial scales with several metrics being used depending on the species' geographic range size, habitat specificity, and local abundance [3]. Although these three components tend to be correlated across species [53], a joint consideration aids in depicting the scales of a species' extinction risk. For instance, species well adapted to a particular habitat may be regionally rare but abundant in appropriate habitat [50],[54]. Considering a range of spatial scales allows evaluation of a range of cases in which climate change, harvesting, or habitat degradation may threaten species 1. Accordingly, we defined two categories of rarity (rare and rarest species) using thresholds and two scales (regional occupancy and local abundance). For the three datasets, regional rare species were defined as those with a regional occupancy of less than 5% of the maximum observed value across the species pool, while the regional rarest species were those with only one occurrence. For reef fish and tropical trees, the local rarest species were defined as those with an average of one individual by sample where present, while for alpine plants the rarest threshold was set at less than 1% of the maximum observed cover (88%), thus at 0.88%. The locally rare species were defined as those with less than 5% of the maximum observed local abundance—that is, those with less than 1.5 individuals by transect for reef fish (using a log scale due to the large magnitude in observed values), less than 4.4% maximum cover for alpine plants, and less than 2.4 individuals by plot for tropical trees.

### Geographic Marginality Estimation

For the three datasets, we estimated species' geographic marginality. For reef fish and tropical trees, the marginality value was calculated as the mean distance from samples where the species occurred to the barycenter of the ecosystem—that is, the geographic center of all the samples. However, this method cannot be applied when the area has an irregular and concave shape since species can be close to the barycenter, thus having a low marginality value, while sampled on the edge. This was the case for the alpine geographic domain. As an alternative, we considered the mean distance from samples where the species occurred to the closest edge of the domain as a measure of geographic marginality. To compare geographic marginality values across ecosystems, those values were standardized by dividing species values by the maximum value observed across species from each ecosystem.

### Functional Indices

#### Functional Distinctiveness

Many distinctiveness measures have been developed recently [55], most of them being designed within a phylogenetic perspective and based on trees linking species. So, as a first step, we used the functional traits to estimate a Gower distance matrix between all species pairs [56],[57]. Then we built the most reliable functional dendrogram linking all species in a functional space [58]—that is, the dendrogram that provides the least distortion between original distances between species pairs and the ultrametric distances on the tree where species are clustered according to their biological traits.

We adapted the Evolutionary Distinctiveness index [59],[60], which measures species' relative contributions to phylogenetic diversity, for use within a functional context. First, for each branch of the functional dendrogram, we estimated a value equal to its length divided by the number of species subtending the branch. The functional distinctiveness of a species is simply the sum of these values for all branches from which the species is descended, to the root of the functional dendrogram.

The estimation of functional distinctiveness was achieved using the R package “*ade4*” and function *originality*. We tested other functional distinctiveness indices (QE and Equal-split index) using this *originality* function, and they were all highly correlated (>0.8). The distribution of functional distinctiveness values for each ecosystem is shown in Figure S2.

#### Functional Insurance And Vulnerability

The insurance value of the function performed by each species *i* (*IV_i_*) was estimated by taking into account the occurrences of the 1% (other thresholds were used with no effect on the results) nearest functional neighbors *j* in the focal assemblage and the functional distances to these neighbor species:where *O_j_* is the number of occurrences of neighbor species *j* and *d_ij_* is the functional distance between species *i* and *j*. *IV_i_* is maximized when species *i* has nearest neighbors that are functionally redundant (*d_ij_* = 0) and common. *IV_i_* decreases when either the functional nearest neighbors are distant from the focal species—that is, decreasing redundancy for that function (*d_ij_* increases)—or when the functional nearest neighbors have low numbers of occurrences. The exponential was used to avoid high weights from far species with high abundances in the estimation of insurance. We make the assumption that redundancy is mainly carried by the closest neighbors in the functional space. Since the abundance of the focal species is not taken into account in *IV_i_* calculation, there is no bias or circularity—that is, common species are not expected to have more insurance than rare species.

We introduced a new index of functional vulnerability (*FV*) that is inversely proportional to insurance, standardized between 0 and 1 and weakly influenced by the high magnitude of insurance values obtained among species. *FV_i_* was calculated as:where max(*IV*) and min(*IV*) are the maximum and minimum functional insurance value across the pool of species, respectively. *FV_i_* increases when the functional insurance decreases with a maximum of 1 when *IV_i_* = min(*IV*) and a minimum of 0 when *IV_i_* = max(*IV*).The distribution of functional vulnerability values for each ecosystem is shown in Figure S2.

We used the R package “*FNN*” to identify the 1% nearest neighbors from the Gower distance matrix.

### Statistical Analyses

The relationship between the commonness of species over the region and their functional distinctiveness—that is, how different a species is from the other species in the assemblage in terms of ecologically significant functional traits—is triangular, with a weak relationship between the means of the two variables, and the variance of the response variable changes with values of the independent variable in all three ecosystems. Since conventional regression-correlation analyses are inappropriate for testing such relationships, we performed, in addition to classical ordinary least square regressions, quantile regressions (95^th^ and 99^th^ quantiles) that are able to detect constraints of an independent variable on the upper limit of a response variable while assuming a linear relationship between the maximum possible value of a response variable along the gradient of the independent variable [61].

We used the *rq* function from the *quantreg* package to build quantile regressions. Confidence intervals for each quantile regression were obtained using a kernel estimate implemented in the function *summary*.

To test whether rare and rarest species were disproportionately represented along the gradient in functional vulnerability, we classified species by their degree of functional vulnerability (High, [0–0.05]; Moderate, [0.05–0.25]; Low, [0.25–0.5]; Least, [0.5–1]). We chose this irregular binning to focus on species supporting the most vulnerable functions—that is, those of primary conservation concern—in the same vein as the classification of biodiversity hotspots focuses on the top 5% regions. Then we implemented a geometric series (0.25, 0.5, and 1) to define the other thresholds in order to discriminate species with a moderate degree of functional vulnerability—that is, those with a medium conservation concern—from the others—that is, with a low or very low conservation concern, without inflating the number of categories. For each level, we observed the percentage of rare and rarest species. We then randomized species among functional vulnerability levels (without replacement) to test whether the observed percentages were greater or less than expected using unilateral thresholds (5% and 95%) given the patterns observed in Figure 1.

To test whether the level of species geographic marginality was similar among functional vulnerability levels as previously defined, we first calculated the observed mean species marginality by level. We then used a first null model where marginality values were randomly distributed among all species to test whether the observed means were greater or less than expected by chance using unilateral thresholds (5% and 95%) given the patterns observed in Figure 3. Indeed, common species, which are underrepresented among species supporting highly and moderately vulnerable functions, cannot have high marginality values as present in many samples over the ecosystem, while rare species, which are overrepresented in those functional vulnerability levels, are more likely to be marginal. Since this test is highly conservative and does not account for the distribution of commonness among functional vulnerability levels, we implemented a second null model where we removed common species (those with a commonness value higher than the median) and where we shuffled marginality values among uncommon species from different functional vulnerability levels.

We provided the number of species in each category (rare, rarest, functional vulnerability levels), for each ecosystem and for each statistical test in Table S1.

## Supporting Information

Figure S1 Mean species geographic marginality for different levels of functional vulnerability. The functional vulnerability index is scaled (0–1) and was divided into four categories from high to least. For each level of vulnerability we obtained the confidence interval at 90% (grey horizontal bar) by randomization and we tested whether the mean species geographic marginality is higher (red star) or lower (blue star) than expected by chance, otherwise indicated by a black circle. The vertical grey line is the median obtained at random. We used two null models: in the first one, marginality values were shuffled among all species (upper panels), while in the second one, we excluded the 50% most common species (lower panels) before shuffling marginality values. Indeed ubiquitous species cannot have high marginality values because they occur in many samples over the ecosystem and thus bias the results towards higher marginality values for functional vulnerability levels with more rare species. The lower panel is thus the better test of the hypothesis.
(TIF)

Figure S2 Distribution of functional distinctiveness and functional vulnerability values for 846 coral reef fishes, 2,979 alpine plants, and 662 tropical trees. Functional distinctiveness, expressed as a proportion of the maximum observed value, quantifies the uniqueness of species biological traits from the rest of the pool in the ecosystem. Functional vulnerability, scaled between 0 and 1, quantifies the lack of functional insurance provided by the rest of the pool to the focal species in terms of functional traits and regional occupancy.
(TIF)

Table S1 Number of species for each statistical analysis, each scale, each dataset, and each functional vulnerability level if any.
(XLSX)

Text S1 List and details of functional traits for fish, plants, and trees.
(DOC)
