# Supplementary figures and images for: Rare Species Support Vulnerable Functions in High-Diversity Ecosystems
Source: PLoS Biol. 2013 May 28;11(5):e1001569. doi: 10.1371/journal.pbio.1001569 (PMC3665844; doi:10.1371/journal.pbio.1001569)

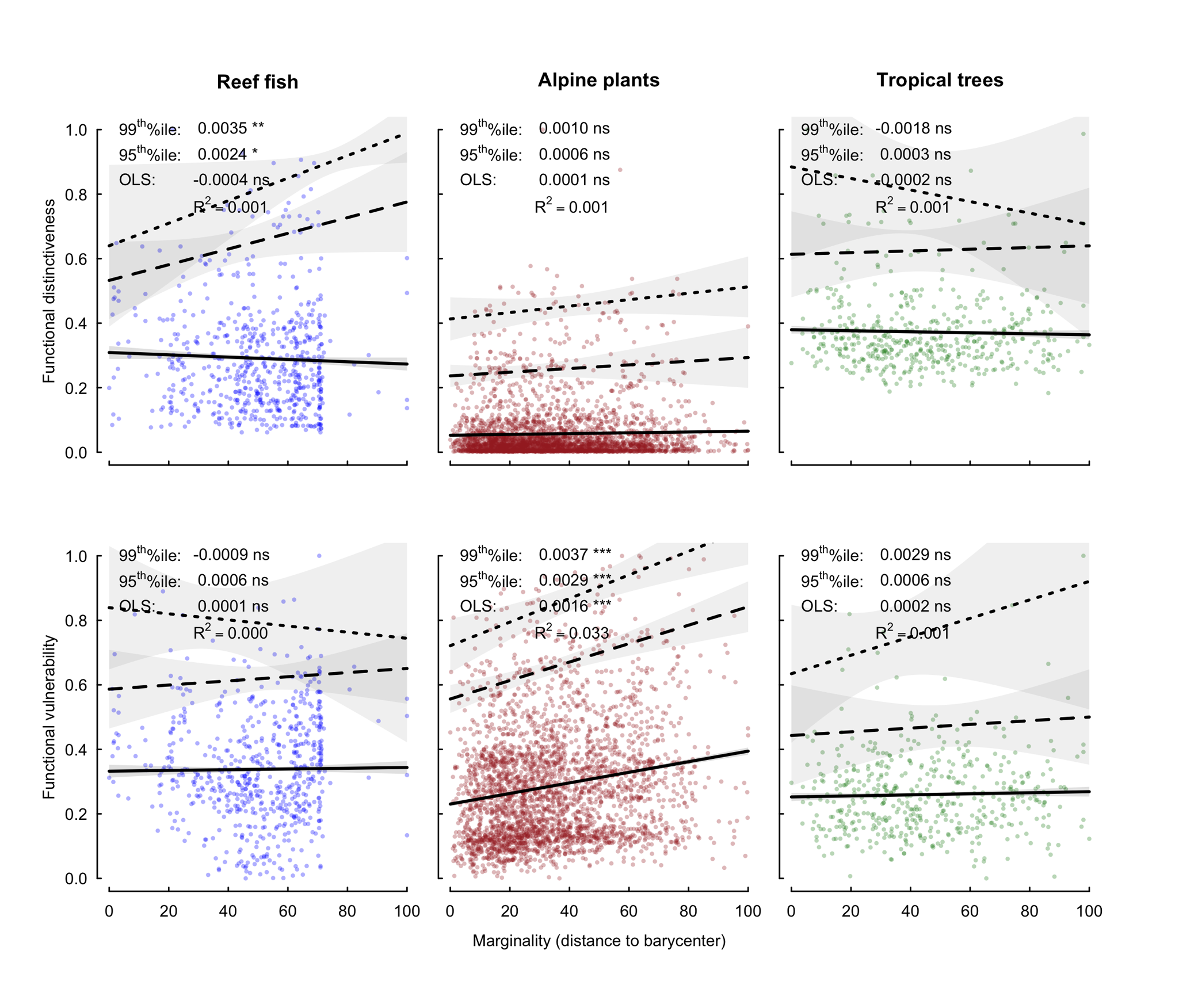

Supplement: Figure S1 — Mean species geographic marginality for different levels of functional vulnerability. The functional vulnerability index is scaled (0–1) and was divided into four categories from high to least. For each level of vulnerability we obtained the confidence interval at 90% (grey horizontal bar) by randomization and we tested whether the mean species geographic marginality is higher (red star) or lower (blue star) than expected by chance, otherwise indicated by a black circle. The vertical grey line is the median obtained at random. We used two null models: in the first one, marginality values were shuffled among all species (upper panels), while in the second one, we excluded the 50% most common species (lower panels) before shuffling marginality values. Indeed ubiquitous species cannot have high marginality values because they occur in many samples over the ecosystem and thus bias the results towards higher marginality values for functional vulnerability levels with more rare species. The lower panel is thus the better test of the hypothesis. (TIF) [file pbio.1001569.s001.tif]

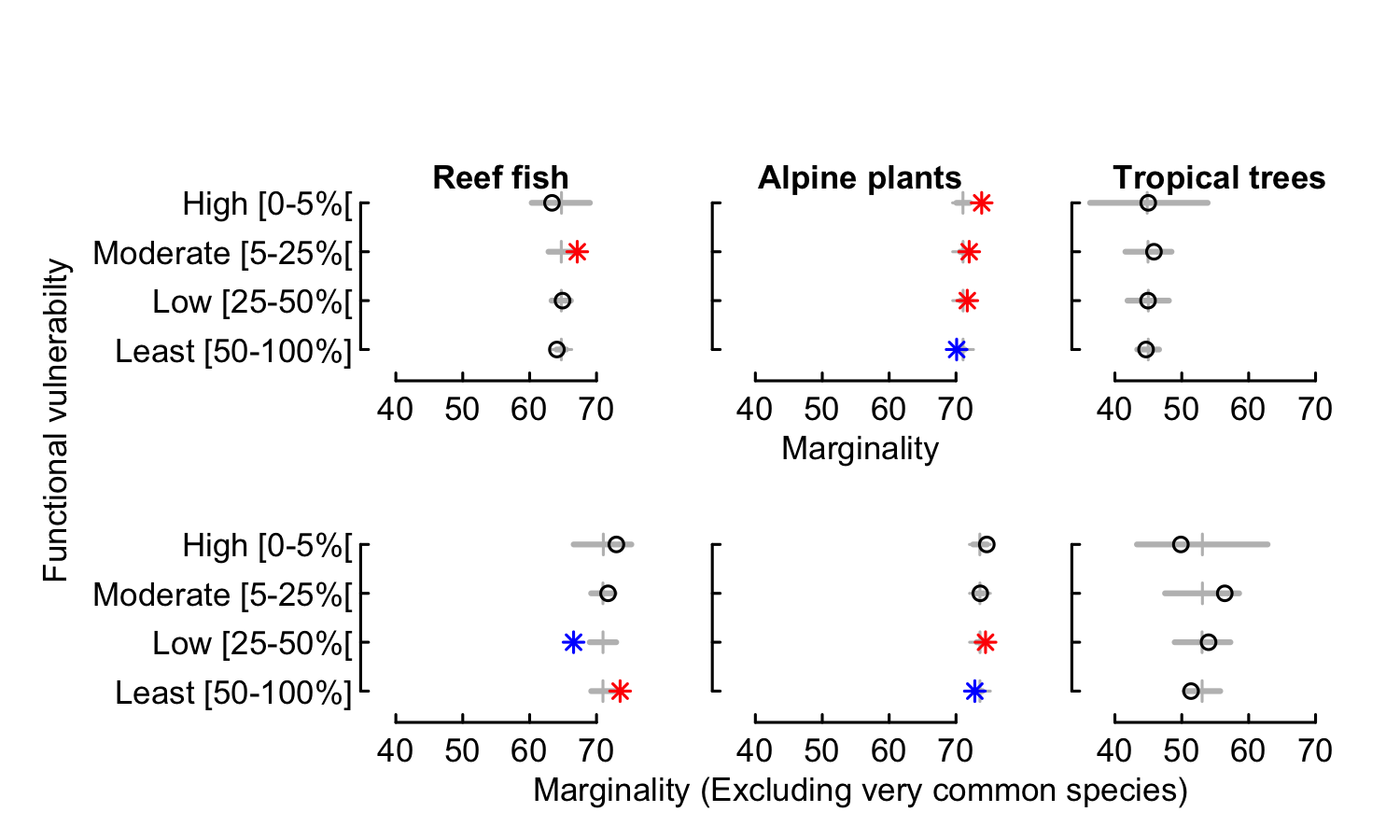

Supplement: Figure S2 — Distribution of functional distinctiveness and functional vulnerability values for 846 coral reef fishes, 2,979 alpine plants, and 662 tropical trees. Functional distinctiveness, expressed as a proportion of the maximum observed value, quantifies the uniqueness of species biological traits from the rest of the pool in the ecosystem. Functional vulnerability, scaled between 0 and 1, quantifies the lack of functional insurance provided by the rest of the pool to the focal species in terms of functional traits and regional occupancy. (TIF) [file pbio.1001569.s002.tif]
