# Supplementary material for: Rare Species Support Vulnerable Functions in High-Diversity Ecosystems
Source: PLoS Biol. 2013 May 28;11(5):e1001569. doi: 10.1371/journal.pbio.1001569 (PMC3665844; doi:10.1371/journal.pbio.1001569)
Supplement: Text S1 — List and details of functional traits for fish, plants, and trees. (DOC) [file pbio.1001569.s004.doc]

**Rare species support vulnerable functions in high-diversity ecosystems**

**Fish functional traits**

1- Species size: the known maximum size;

2- Diet: the two major known components of the species’ diet were used. Food items were classified into 8 major categories: P: fish or nekton; C1: large benthic invertebrates; C2: small benthic invertebrates; Z: plankton; H1: macro-algae and seagrass; H2: micro-algae (turf), blue-green algae; Co: sessile invertebrates (coral and sponge mainly); D: detritus or unidentified organic matter (either of animal or vegetal origin);

3- Home range: Species were grouped into three categories: S: sedentary species, i.e. species which tend to stay within a very restricted range from less than 1 m² up to a few 100’s m²; M: mobile species, i.e. species which stay for long periods (from several days to several months) within a restricted but rather large (from 100 m² up to several hectares) range; W: wide ranging species that circulate over large to very large extent of reefs and may change from one reef to another within the same day or over a short period of time;

4- Activity: Reef fish species that are active only during the day (D), only during the night (N) or active at all time of day or night (B);

5- Schooling: a- solitary: species which individuals tend to be found single; b- paired: species which are usually found living in pairs. The individuals forming the pair may not necessarily be one by the other at all time (e.g. Chaetodons) but they stay in close contact most of the time; c- small groups: species which stay in small schools or groups (an aggregation of individuals which stay together but do not have a schooling behavior such as swimming all in the same direction at all time), usually less than 20 individuals at a time; d- medium size groups, e.g. species found in schools or groups of 20 to 50 individuals (on average); e- large groups, species forming large schools or groups with usually more than 50 individuals at a time;

6- Level in the water column: Most reef fish species tend to stay at the same level in the water column most of the time. Species were divided into 3 categories:- B: species which stay most of the time right on the bottom; L: species staying just above the bottom, but usually not laying on the bottom; H: species swimming high above the bottom.

**Plant functional traits**

1- Leaf area: the average area of leaves;

2- Leaf dry matter content: dry mass of a leaf divided by its water-saturated fresh mass expressed in mg.g-1;

3- Leaf nitrogen content: the total nitrogen per unit of dry leaf mass expressed in mg.g^-1^;

4- Average vegetative height: the distance between the top of the photo- synthetic tissue and the ground;

5- Seed mass: average seed mass;

6- Specific leaf area: the area of a portion of a leaf by the dry weight of that same portion of leaf;

7- Dispersion mode: dysochory, dysochory-epichory, dysochory-meteorochory, dysochory-endochory, dysochory-hydrochory, dysochory-myrmecochory, epichory, epichory-meteorochory, epichory-hydrochory, epichory-myrmecochory, meteorochory, meteorochory-myrmecochory, anthropochory, anthropochory-boleochory, anthropochory-endochory, anthropochory-myrmecochory, anthropochory-autochory, anthropochory-dysochorya, anthropochory-epichorya, anthropochorymeteorochory, boleochory, boleochory-endochory, boleochory-hydrochory, boleochory-myrmecochory, boleochory-dysochorya, boleochory-epichorya, boleochory-meteorochorya, endochory, endochory-hydrochory, endochory-myrmecochory, endochory-epichorya, endochory-meteorochorya, hydrochory, hydrochory-meteorochorya, myrmecochory, utochory, utochory-dysochory, utochory-epichory, utochory-meteorochory, utochory-boleochory, utochory-endochory, utochory-hydrochory, utochory-myrmecochory;

8- Life span: biennial, biennial- pluriennial, annual, annual-pluriennial, annual-biennial, annual-biennial-pluriennial, pluriennial;

9- Leaf persistence: evergreen, evergreen-partly wintergreen, evergreen-wintergreen, partly-wintergreen, partly wintergreen – wintergreen, wintergreen, deciduous, deciduous - green only from autumn to spring time, deciduous pre-summergreen, deciduous-partly wintergreen, deciduous-wintergreen, green only from autumn to spring time, pre-summergreen, pre-summergreen-wintergreen, without green leaves (full parasites or saprophytes);

10- Woodiness: frutescent, suffrutescent, herbeaceous, herbeaceous- suffrutescent.

**Tree functional traits**

1- Foliar δ13C composition as an estimate of water use efficiency, standardized as residuals of regression with tree size;

2- Foliar carbon:nitrogen ratio;

3- Foliar K concentration in %;

4- Foliar N concentration in %;

5- Laminar chlorophyll content, in µg mm-2;

6- Laminar total surface area;

7- Laminar toughness via punch test, in N;

8- Leaf blade unit (leaflet) surface area;

9- NH4+ utilization, in %, derived from foliar δ15N composition;

10- Specific Leaf Area (SLA);

11- Trunk bark thickness, in mm;

12- Trunk xylem density, in g cm-3;

13- Twig bark thickness, in mm;

14- Twig xylem density, in g cm-3.
